# A protocol to optimize non-invasive brain stimulation for post-stroke rehabilitation

**DOI:** 10.1016/j.mex.2025.103209

**Published:** 2025-02-05

**Authors:** Ayesha Juhi, Manul Das, Dinesh Bhatia, Suman Dhaka, Rajesh Kumar, Deepak Kumar, Shreya Sharma, Pritam Kumar Chaudhary, Chanchal Goyal, Md Asif Khan, Himel Mondal

**Affiliations:** a Department of Physiology, All India Institute of Medical Sciences, Deoghar, Jharkhand, India; b Clinical Research Centre for Neuromodulation in Psychiatry, Central Institute of Psychiatry, Ranchi, Jharkhand, India; c Department of Biomedical Engineering, North-Eastern Hill University, Shillong, Meghalaya, India; d School of Liberal Arts and Center for Brain Science and Application (SAIDE), Indian Institute of Technology, Jodhpur, India; e Department of Internal Medicine, All India Institute of Medical Sciences, Deoghar, Jharkhand, India; f Department of Physical Medicine and Rehabilitation, All India Institute of Medical Sciences, Deoghar, Jharkhand, India; g Neuromodulation Laboratory, Department of Physiology, All India Institute of Medical Sciences, Deoghar, Jharkhand, India; h Centre for Evidence for Guidelines, Indian Council of Medical Research, New Delhi, India; i Descriptive Research Division, Indian Council of Medical Research, New Delhi, India

**Keywords:** Non-invasive brain stimulation, rTMS, tDCS, Cognitive function, Motor function, Transcranial direct current stimulation, Transcranial magnetic stimulation, Stroke rehabilitation, Stroke, Cognition, Brain, Neuromodulation

## Abstract

This randomized controlled trial investigates the optimal dosing for post-stroke rehabilitation using repetitive transcranial magnetic stimulation (rTMS) and transcranial direct current stimulation (tDCS). Previous studies demonstrated improvements in cognitive and motor functions with specific intensities of rTMS and tDCS, but this trial explores various frequencies and currents to optimize therapeutic outcomes. A total of 128 post-stroke patients (within 1–6 months of stroke) with paraplegia or hemiplegia are recruited. Patients are divided into four groups for both rTMS (n = 49) and tDCS (n = 49): three groups with different stimulation intensities (1 Hz, 5 Hz, 10 Hz for rTMS; 0.5 mA, 1 mA, 2 mA for tDCS) and a sham control group. Along with this, there is a standard therapy group (n = 30) as control. Participants receive 20 min sessions, five days a week, over six weeks. Cognitive and motor assessments are conducted at 4 weeks, 6 weeks, and 6 months to measure short-term and sustained effects.

• Hemodynamically stable post-stroke patients randomized in four groups in rTMS and tDCS each and their baseline cognitive and motor function assessed
• Application of the two types of therapy for 6 weeks
• Checking improvement of cognitive and motor function and compare the improvement among subgroups of recipient of various frequencies and currents

## Graphical abstract

Optimizing post-stroke rehabilitation by testing different intensities of rTMS and tDCS to enhance cognitive and motor recovery.

**Figure:**
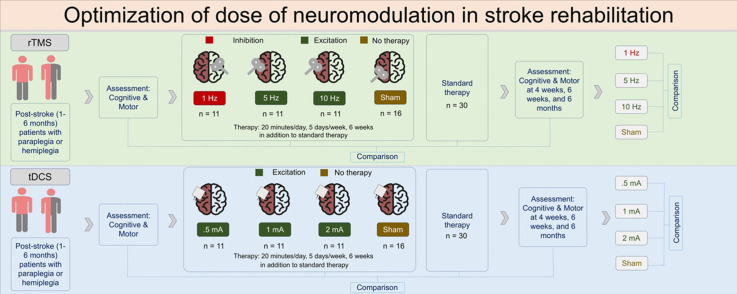

Specifications table

**Table:** 

Subject area:	Neuroscience
More specific subject area:	Rehabilitation
Name of your protocol:	Neuromodulation
Reagents/tools:	(1) Montreal Cognitive Assessment (2) Fugl Meyer Assessment (3) Transcranial magnetic stimulation machine, Neuro-MS/D, Neurosoft LLC, Russia (4) High-definition transcranial direct current stimulation machine, Neurostim, Neurosoft LLC, Russia
Experimental design:	Double blinded randomized controlled trial
Trial registration:	CTRI/2024/05/*066729* registered at https://ctri.nic.in
Ethics:	Approved by Institutional Ethics Committee (2023–130-EMP-03). Patients will be recruited after taking written consent for voluntary participation.
Value of the Protocol:	Optimizes brain stimulation parameters (frequencies for rTMS and currents for tDCS) to enhance cognitive and motor recovery in stroke patients

## Background

Stroke is a major health concern worldwide, with its devastating effects leading to significant physical and cognitive disabilities in about 50 % survivors [1]. The journey toward recovery is long and challenging, requiring comprehensive rehabilitation strategies that aim to restore as much function as possible [2]. While various rehabilitation methods exist [3,4], there remains a search for more effective techniques that address both motor and cognitive deficits.

In recent years, non-invasive brain stimulation (NIBS) techniques, such as repetitive transcranial magnetic stimulation (rTMS) and transcranial direct current stimulation (tDCS), have gained attention for their potential to enhance stroke recovery [5,6]. These techniques use magnetic fields or weak electrical currents to modulate brain activity, promoting neuroplasticity—the brain's ability to reorganize itself after injury [7,8]. Neuroplasticity is crucial for recovery, as it allows the brain to form new neural connections and compensate for the damaged areas [9]. By stimulating or inhibiting specific regions of the brain associated with motor control or cognition, NIBS can enhance motor and cognitive functions [10].

Several studies highlight the potential of non-invasive brain stimulation techniques for stroke rehabilitation. Liu et al. demonstrated that 10 Hz TMS could improve daily living and attention functions, though long-term effects remain unexplored [11]. Yin et al. applied high-frequency rTMS to the left dorsal lateral prefrontal cortex, observing cognitive improvements without follow-up to assess lasting benefits [12]. A separate trial combining 5 Hz rTMS and cognitive training reported potential gains in cognition despite high dropout rates [13]. In contrast, low-frequency rTMS appeared more effective for motor and neurological recovery in ischemic stroke patients compared to high-frequency stimulation [14].

Studies on tDCS have also reported positive effects. Yan et al. found that tDCS benefits were more pronounced in patients with shorter disease durations, stressing the need for standardized protocols [15]. Research on bihemispheric tDCS combined with physical therapy indicated improvements in upper limb function, yet issues like lesion heterogeneity suggest that larger, long-term studies are required [16]. Despite promising outcomes, larger, blinded RCTs with better recruitment strategies are necessary to compare rTMS and tDCS efficacy and establish generalizability [17].

Despite the promise shown by NIBS in stroke rehabilitation [18], there is need of consensus on optimal frequencies and current for patients [19]. Research has demonstrated that factors such as frequency, intensity, and duration of stimulation play critical roles in determining the efficacy of these techniques [20]. However, the optimal combination of these parameters remains unclear, leading to inconsistent results across studies.

With this background, this study protocol was prepared for a clinical trial to address these critical gaps by exploring the optimization of rTMS and tDCS parameters in stroke rehabilitation.

## Description of protocol

### Patient allocation

Before the intervention, all participants undergo a comprehensive assessment to evaluate their baseline cognitive and motor functions. The cognitive assessment will be conducted using the Montreal Cognitive Assessment (MoCA) test, designed to measure a range of cognitive abilities such as memory, attention, and language skills. Motor function will be assessed using the Fugl-Meyer Assessment (FMA), which evaluates sensorimotor function in stroke patients. These baseline measurements are essential to determine each participant's starting level of impairment and to facilitate comparison with post-therapy outcomes.

**Fig. 1:**
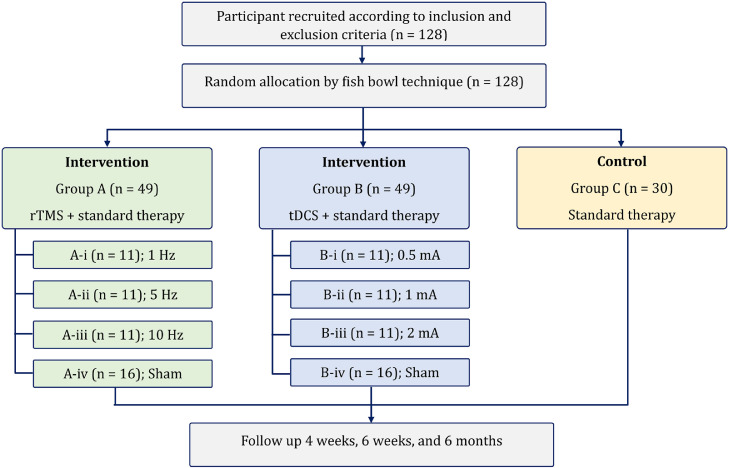
Patient randomization and allocation.

Participants will be randomly assigned to one of three groups: (A) rTMS with standard therapy, (B) tDCS with standard therapy, or (C) standard therapy as the control group. The random allocation is shown Fig. 1. The patients in the active therapy group will receive the therapy with the pre-fixed protocol and patients in sham group will be provided with all settings of therapy with same time, but in a sham coil orientation for rTMS and 0.5 mA for initial and end 60 s with zero current in between for tDCS. The control group will receive only physiotherapy as the standard stroke rehabilitation therapy.

To achieve this, researchers employ a randomized selection process using identically folded chits marked with group codes (e.g., a chit A-iii will allocate the patient to active rTMS of 10 Hz). Each participant selects a chit from a bowl, ensuring an unbiased distribution across groups and avoiding selection bias.

### Therapy

#### Active rTMS group

**Fig. 2:**
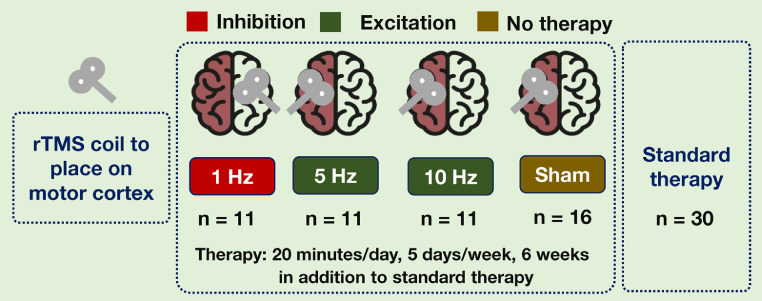
Therapy intensity for repeated transcranial magnetic stimulation.

This group receives repetitive transcranial magnetic stimulation (rTMS), where magnetic pulses target the motor cortex. The intervention follows three frequency settings (1 Hz, 5 Hz, and 10 Hz) to identify the optimal dose for effectiveness. The 1 Hz therapy will be provided on the contralateral side of the affected side while other will be provided on the affected side (Fig. 2).

Low-frequency magnetic stimulation, typically <5 Hz, is known to reduce cortical excitability, producing an inhibitory effect on brain activity. The 5 Hz and 10 Hz therapy will be provided on the affected side of the cortex. Higher frequencies of magnetic stimulation, such as 5 Hz and 10 Hz, are considered to have excitatory effects on brain activity, increasing cortical excitability. In sham therapy, the coil will be placed on the side of the affected cortex.

**Fig. 3:**
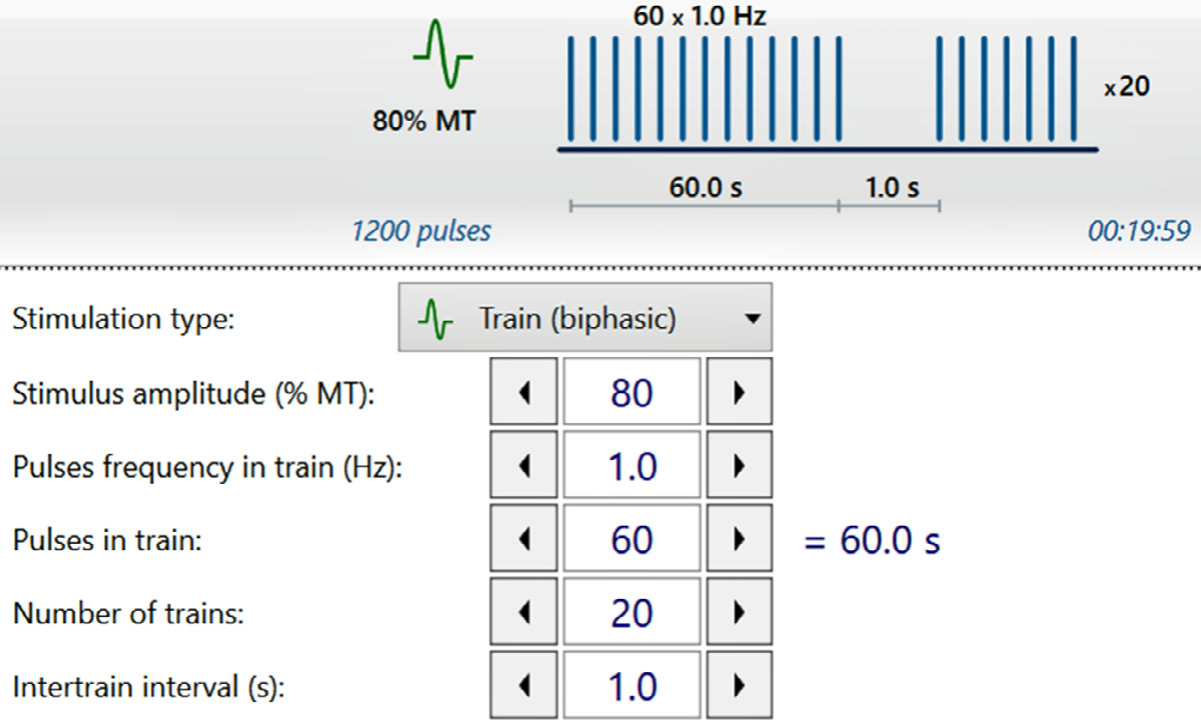
First rTMS protocol showing the intensity, pulses, and trains.

At the beginning of the study, the resting motor threshold (RMT) of each participant will be established using the software's calibration tools. The RMT is the lowest stimulation intensity at which thumb movements are observed in at least 5 out of 10 pulses delivered to the contralateral motor hotspot. Then, the stimulation frequency, intensity (80 % of the MT), and train duration will be selected as per the prefixed protocol. The first therapy protocol is shown in Fig. 3, and the rest of the protocol is shown in Table 1.

**Table 1:** Details of the protocols for the intervention group (Group A) of rTMS.

Frequency (Hz)	Intensity (% of RMT)	Session duration (min)	Train duration (seconds/train)	Inter-train interval (if any, sec)	Number of trains per session	Number of sessions	Number of pulses/sessions	Stimulation site
1	80	20	1200	Continuous	1	30	1200	Ipsilateral
5	80	20	10	20	40	30	2000	Contralateral
10	80	20	5	25	40	30	2000	Contralateral

RMT: Resting motor threshold.

#### Sham rTMS group

**Fig. 4:**
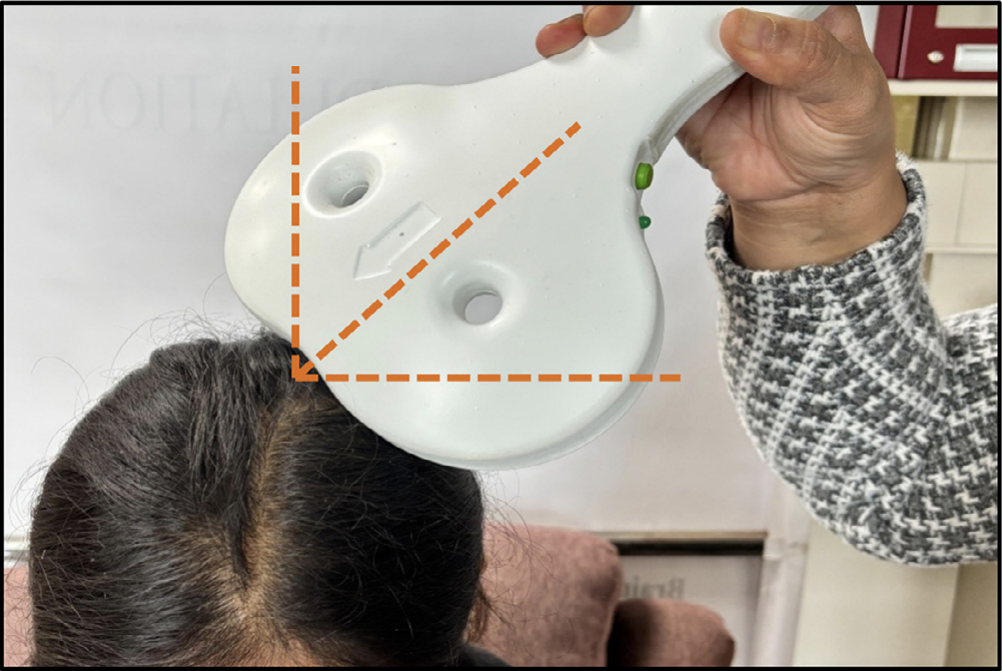
rTMS coil position in sham treatment.

For sham stimulation, the figure-of-eight coil can be tilted 45° from the scalp, with both wings touching the head (Fig. 4) [21]. This orientation minimizes cortical stimulation, prevents the induction of motor-evoked potentials (MEPs), and effectively replicates the sensory and auditory effects of active TMS.

#### Active tDCS group

**Fig. 5:**
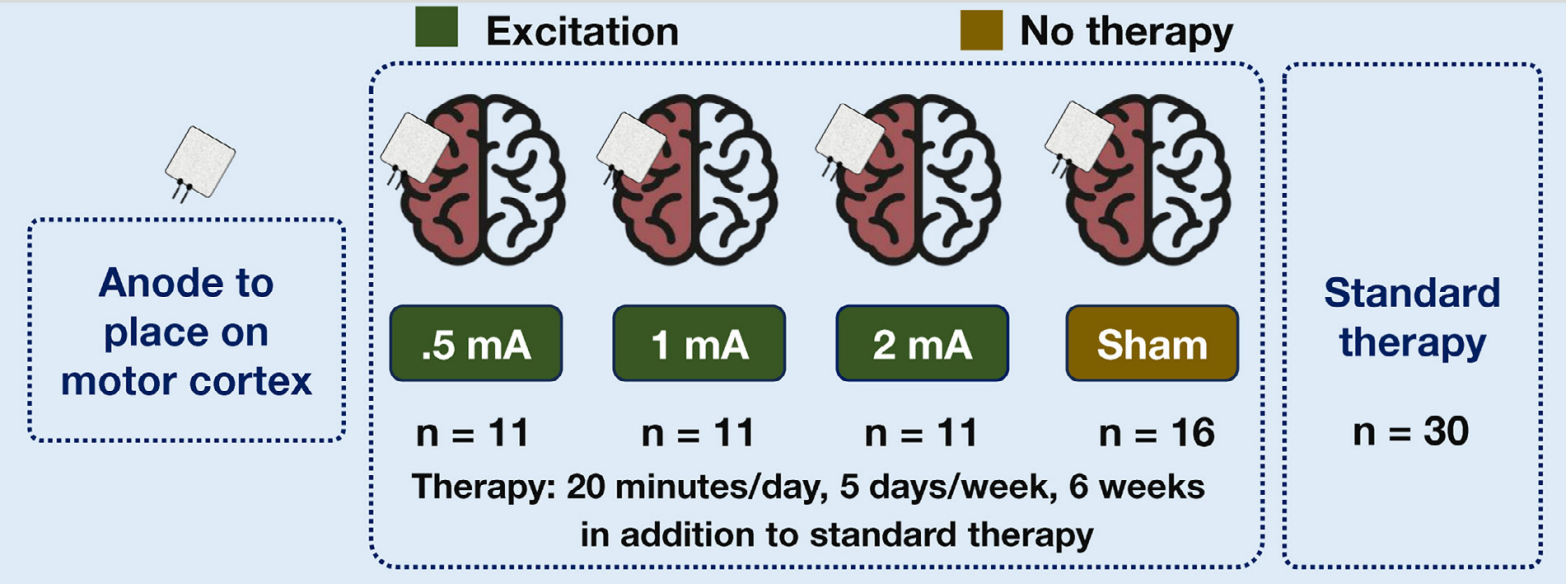
Therapy intensity for transcranial direct current stimulation.

Participants receive conventional transcranial direct current stimulation (tDCS) on the motor cortex. For the therapy, the anode is placed on the primary motor cortex on the lesioned side, and the cathode is placed on the contra-lateral supraorbital area. The dosage settings vary in current intensity (0.5 mA, 1 mA, and 2 mA), as shown in Fig. 5.

**Fig. 6:**
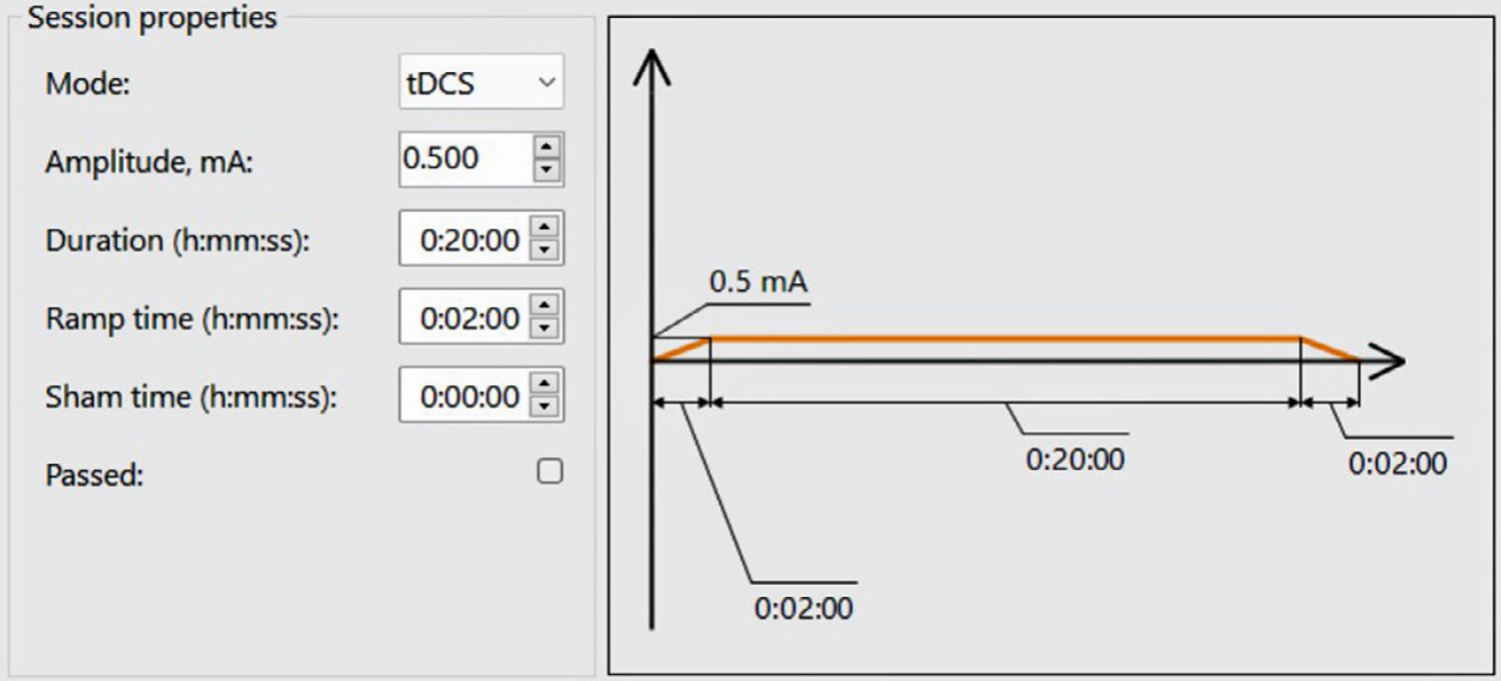
The amplitude, duration, and ramp time of first therapy protocol of active tDCS.

The software interface for the first therapy is shown in Fig. 6. In this protocol, the therapy will be provided with an amplitude of 0.5 mA for 20 min. The current will be provided with a ramp up and ramp down of two minutes in initial and ending of the time, respectively.

The patient's scalp is prepared for electrode placement by slightly rubbing the scalp with electrode gel, and the electrodes are encased in 0.9 % saline-soaked sleeves. These sleeve-enclosed electrodes are placed on the scalp as close as possible and fastened by rubber fasteners. If the patient is having a lesion on the left hemisphere and the anode is placed on the left primary motor cortex area. The cathode is placed on the supraorbital area on the right side. The therapist can set the protocol on the controlling computer when the tDCS device is connected to the computer by a USB. The device can also be operated from the user interface of the device itself (having control keys and display) when the computer is not available.

#### Sham tDCS group

The sham tDCS group will be provided with the electrode placed exactly on the motor cortex and supraorbital region with the application of a saline-soaked sleeve of electrode just like the active group. A current of 0.5 mA will be applied for the initial and the final 60 s and zero current will be delivered for the rest of the session. This approach ensures that participants perceive the initial sensations associated with tDCS, such as tingling or itching, thereby maintaining blinding in controlled studies.

For all the active and sham groups, the session will last for 20 min, five days a week, across six weeks. This structured schedule allows for consistent and comparable intervention times across all groups.

#### Standard therapy group

This group will receive only physiotherapy as the standard stroke rehabilitation therapy. This group will be serving as the control group of the study. Physiotherapy includes a combination of functional, aerobic, and resistance exercises 20 min. In addition to this, the drug therapy as prescribed by the treating physician will be continued by the patients.

#### Post-therapy assessment

During and following the intervention phase, participants undergo the same assessments (MoCA and FMA) as performed in the pre-therapy stage. These assessments will occur immediately after therapy, at four weeks mid-intervention, after completion of six weeks, and again at six months post-intervention to evaluate both short- and long-term effects. This sequence will enable to monitor any improvements, non-improvement, or declines in cognitive and motor functions over time.

#### Dosage comparison

In both the rTMS and tDCS groups, the intervention is applied at three different dosage levels to identify the most effective parameters for enhancing cognitive and motor outcomes. The response to each dosage is compared across groups, considering factors such as accuracy of motor function improvement, cognitive gains, and patient tolerance. This comparative approach aims to optimize the therapeutic dose, balancing efficacy with safety. If a lower frequency or amplitude is found to be equally effective to a higher frequency or amplitude, then the study will conclude by recommending a lower frequency or amplitude.

## Limitations

This protocol has some limitations beyond our capability to adjust. Patient responses to non-invasive brain stimulation (NIBS) techniques like rTMS and tDCS can vary widely due to differences in brain structure, stroke severity, and neural plasticity. This variability can make it difficult to generalize optimal parameters across a diverse population. Additionally, the strict inclusion and exclusion criteria used to select participants may limit generalizability, as individuals with more severe impairments or certain comorbidities might respond differently to the treatment. Furthermore, the study uses fixed dosing parameters (frequencies and intensities) for rTMS and tDCS, which might not be optimal for each patient. Although necessary for research consistency, the lack of individualized dosing could limit the intervention's overall efficacy. Additionally, while the six-week intervention duration and single six-month follow-up provide insights into short- and medium-term effects, a longer follow-up period would be needed to determine lasting benefits. However, we are limited by the logistics of the study.

## CRediT authorship contribution statement

**Ayesha Juhi:** Conceptualization, Methodology, Resources, Project administration, Supervision, Writing – review & editing. **Manul Das:** Methodology, Supervision, Writing – review & editing. **Dinesh Bhatia:** Conceptualization, Methodology, Supervision, Writing – review & editing. **Suman Dhaka:** Conceptualization, Methodology, Supervision, Writing – review & editing. **Rajesh Kumar:** Conceptualization, Methodology, Supervision, Writing – review & editing. **Deepak Kumar:** Conceptualization, Methodology, Supervision, Writing – review & editing. **Shreya Sharma:** Methodology, Data curation, Software, Writing – review & editing. **Pritam Kumar Chaudhary:** Methodology, Data curation, Software, Writing – review & editing. **Chanchal Goyal:** Methodology, Supervision, Writing – review & editing. **Md Asif Khan:** Methodology, Supervision, Writing – review & editing. **Himel Mondal:** Conceptualization, Methodology, Software, Resources, Visualization, Writing – original draft, Writing – review & editing.

## Declaration of competing interest

The authors declare that they have no known competing financial interests or personal relationships that could have appeared to influence the work reported in this paper.

## Data Availability

No data was used for the research described in the article.
